# Immunodeficiency with susceptibility to lymphoma with complex genotype affecting energy metabolism (*FBP1, ACAD9)* and vesicle trafficking *(RAB27A)*

**DOI:** 10.3389/fimmu.2023.1151166

**Published:** 2023-06-14

**Authors:** Nina Brauer, Yuto Maruta, Miriam Lisci, Katharina Strege, Ilske Oschlies, Hikari Nakamura, Svea Böhm, Kai Lehmberg, Leon Brandhoff, Stephan Ehl, Nima Parvaneh, Wolfram Klapper, Mitsunori Fukuda, Gillian M. Griffiths, Hans Christian Hennies, Tim Niehues, Sandra Ammann

**Affiliations:** ^1^Department of Pediatrics, Helios Klinikum, Krefeld, Germany; ^2^Department of Integrative Life Sciences, Graduate School of Life Sciences, Tohoku University, Sendai, Japan; ^3^Department of Medicine, Cambridge Institute for Medical Research, University of Cambridge, Cambridge, United Kingdom; ^4^Department of Immunobiology, University of Lausanne, Epalinges, Switzerland; ^5^Department of Pathology, Haematopathology Section and Lymph Node Registry, University Hospitals Schleswig-Holstein, Christian-Albrecht University, Kiel, Germany; ^6^Division of Pediatric Stem Cell Transplantation and Immunology, Clinic of Pediatric Hematology and Oncology, University Medical Center Hamburg Eppendorf, Hamburg, Germany; ^7^Cologne Center for Genomics, University Hospital Cologne, Cologne, Germany; ^8^Institute for Immunodeficiency, Center for Chronic Immunodeficiency, Medical Center – University of Freiburg, Faculty of Medicine, University of Freiburg, Freiburg, Germany; ^9^Division of Allergy and Clinical Immunology, Department of Pediatrics, Tehran University of Medical Sciences, Tehran, Iran; ^10^Department of Biological and Geographical Sciences, University of Huddersfield, Huddersfield, United Kingdom

**Keywords:** lymphoma, RAB27A-deficiency, Griscelli syndrome type 2, Epstein-Barr virus, metabolic diseases

## Abstract

**Introduction:**

Inborn errors of immunity (IEI) are characterized by a dysfunction of the immune system leading to increased susceptibility to infections, impaired immune regulation and cancer. We present a unique consanguineous family with a history of Hodgkin lymphoma, impaired EBV control and a late onset hemophagocytic lymphohistiocytosis (HLH).

**Methods and results:**

Overall, family members presented with variable impairment of NK cell and cytotoxic T cell degranulation and cytotoxicity. Exome sequencing identified homozygous variants in *RAB27A*, *FBP1 (*Fructose-1,6-bisphosphatase 1*)* and *ACAD9 (*Acyl-CoA dehydrogenase family member 9*).* Variants in *RAB27A* lead to Griscelli syndrome type 2, hypopigmentation and HLH predisposition.

**Discussion:**

Lymphoma is frequently seen in patients with hypomorphic mutations of genes predisposing to HLH. We hypothesize that the variants in *FBP1* and *ACAD9* might aggravate the clinical and immune phenotype, influence serial killing and lytic granule polarization by CD8 T cells. Understanding of the interplay between the multiple variants identified by whole exome sequencing (WES) is essential for correct interpretation of the immune phenotype and important for critical treatment decisions.

## Introduction

Inborn errors of immunity (IEI) are usually caused by pathogenic gene variants affecting single genes. So far, a total of 485 IEIs have been reported by the International Union of Immunological Societies Expert Committee, resulting in a variety of manifestations, including susceptibility to infections, autoimmunity, impaired immunity, autoinflammation, allergies, bone marrow failure, and occasionally malignancies (1). Comprehensive genetic investigations using whole exome or whole genome analysis sometimes reveal more than one affected gene. In particular, many homozygous gene variants are often found in families with consanguinity. The diagnostic work up and treatment of such patients is challenging.

Increasing numbers of IEIs with the clinical presentation of hemophagocytic lymphohistiocytosis (HLH) have been described in the last decade, including familial HLH (FHL-2-5), Chediak-Higashi syndrome (CHS), Griscelli syndrome type 2 (GS2) or X-linked proliferative-syndrome XLP1 and 2 (1). Primary HLH is a hyperinflammatory disorder, characterized by hyperactivation of macrophages and lymphocytes, predominantly caused by impaired cytotoxic function of NK and CD8 T cells (2). In most cases (about 80%), no external trigger for disease development can be found (3, 4). If an infectious trigger is identified, Epstein-Barr virus (EBV) and cytomegalovirus (CMV) are most common (5, 6). Susceptibility to severe EBV infection and often consequential lymphoproliferative conditions are described for a variety of IEIs with immune dysregulation, among them are XLP1+2, ITK, CD70 or CD27 deficiency. GS2 is caused by mutations in the gene encoding for RAB27A, a member of the Rab GTPase family (7, 8). RAB27A is important for intracellular vesicle transport and interacts with MUNC13-4 to facilitate lytic granule exocytosis at the immunological synapse of NK and CD8 T cells (8–10). Perturbed interaction of RAB27A and MUNC13-4 leads to impaired release of lytic granules and impaired cytotoxic effector function (11). Additionally, in melanocytes RAB27A interacts with melanophilin and the actin-based motor myosin Va and is important for melanosome transport in hair and skin (12). Hair hypopigmentation is common in GS2 patients, but some hypomorphic mutations only interfere with the MUNC13-4 interaction and pigmentation of hair and skin remains normal (13–16). Rare cases of lymphoma in patients with hypomorphic mutations in RAB27A have been reported (15, 16).

Many alterations in genes encoding for proteins involved in the cytotoxic machinery causing HLH pre-disposition have been described, however, the effects of metabolic disorders on the cytotoxic function of NK and CD8 T cell are yet not fully understood.

Inborn errors of metabolism (IEM) are disorders caused by mutations in proteins important for breakdown of nutrients and production of energy, including acyl-CoA dehydrogenase 9 (ACAD9) and fructose-1,6-bisphosphatase (FBP1). Both have high morbidity and mortality, especially during infancy (17–21).

ACAD9 belongs to the family of flavoenzymes, which are important for fatty acid β-oxidation and assembly of the mitochondrial respiratory chain complex I (20, 22). Compound heterozygous mutations affect both functions of ACAD9 and may increase disease severity. ACAD9 is highly expressed in cardiac and skeletal muscles, brain, kidney, and liver. Null-mutations of ACAD9 are associated with embryonic death in mice and no human case with complete loss of ACAD9 has been reported (21, 23–26). ACAD9 deficient patients present with hypertrophic cardiomyopathy, lactic acidosis, hyperglycemia and impaired muscle strength (20, 21).

FBP1 is important during gluconeogenesis in the liver and kidney by converting fructose-1,6-bisphosphate into fructose-6-phosphate (27, 28). FBP1 deficiency leads to hypoglycaemia and lactic acidosis during catabolic episodes, induced by prolonged fasting during infections, high fever or excessive fructose consumption indicated by following lab findings: hyperalaninemia, hyperketonemia, elevetaed ratio of lactate/pyruvate, increased plasma concentration of uric acid, glyceroluria and/or pseudo-hypertriglyceridemia (29–32). Clinical symptoms include abdominal pain, vomiting, irritability, hypervenitilation, apnea, somnolescence, coma or seizures (27, 33, 34). In neither ACAD9 nor FBP1 deficiency has a functional impairment of the immune system been described.

In this family, we investigated the role of a new hypomorphic *RAB27A* variant, on a metabolic disease background, for its pigment distribution in melanocytes, binding to its interaction partners MLPH/SLAC2-A, SLP2-A and MUNC13-4 and its impact on cytotoxic function of NK and T cells.

## Material and methods

### Patients

Written informed consent was obtained from all participants and the study was approved by the institutional review board (University of Freiburg ethics committee’s protocol numbers 143/12 and 40/08).

### Genetic analysis

Whole exome sequencing was performed with samples from patients VI and VIII (**Table 1**). Three μg DNA was fragmented using sonication technology (Diagenode, Seraing, Belgium). The fragments were end repaired and adaptor ligated. Library enrichment process was done by using the Nimblegen SeqCap EZ Human Exome Library v2.0 (Roche, Madison,WI) enrichment kit and sequenced with the Illumina HiSeq2000 instrument (Illumina, San Diego, CA). Data analysis of reads passing filter was done with Burrows-Wheeler Aligner-short in combination with Genome Analysis Toolkit and SAMtools as implemented in Varbank (Cologne Center for Genomics). In-house scripts were applied for filtering against dbSNP, the 1000 Genomes Project, the gnomAD database and an internal database of exome variants. Rare (<1% MAF) missense, nonsense, frameshift, and splice site mutations were taken into account assuming autosomal recessive inheritance.

**Table 1:** Summary of clinical phenotype & genetic mutations.

PAT	Mutation		EBV Immune status	Lymphoma	HLH	Metabolic Syndrome
III	nd					abdominal pain, lactate acidosis
IV	nd		BAL: 16469 copies/μg	yes		no
V	*RAB27A ^+/m^* *(not analyzed: ACAD9^+/m^* *FBP1)*		6920 IU/ml	no		mildly increased creatine kinase
VI	*RAB27A ^m/m^* *ACAD9 ^m/m^* *(not analyzed: FBP1)*		1620 IU/ml	yes		catabolic episodes
VII	*RAB27A ^+/m^* *(not analyzed: ACAD9^+/m^* *FBP1)*		no	no		mildly increased creatine kinase
VIII	*RAB27A ^m/m^* *FBP1 ^m/m^* *ACAD9 ^m/m^*		no	no	partial	increased creatine kinase and myopathy, abdominal pain and hypoglycemia, lactate acidosis
IX	*RAB27A ^m/m^* *FBP1 ^m/m^* *ACAD9 ^+/m^*		14400 IU/ml	chronic-hypoplastic tonsilitis		abdominal pain and hypoglycemia, lactate acidosis

nd, not done; m/m, mutated/mutated (homozygote); +/m = wild type/mutated (heterozygote).

### NK and T cell degranulation

NK or T cell degranulation was performed from fresh PBMC or cultured PHA/IL-2 blasts as described previously (35). For analysis, CD3−CD56+ NK cells were gated and assessed for surface expression of CD107a (BD Pharmingen). The difference between the percentage of NK cells expressing surface CD107a after K562 stimulation and after incubation with medium alone (ΔCD107a). For analysis of CTL degranulation, CD3+CD8+ T cells were stimulated with anti-CD3/CD28 activation beads (dynabeads) and difference in the mean fluorescence intensity (MFI) of CD107a expression was compared with the unstimulated control sample. Cells were analyzed with flow cytometry (Navios flow cytometer, Beckman Coulter) and FlowJo software.

### Cytotoxic T cell line

Peripheral blood mononuclear cells (PBMCs) or lymphocytes from tonsil sample (patient IX) were stimulated with PHA (1.25 μg/mL) and ∼100 U/mL human IL-2 (produced from IL-2 expressing X63 myeloma cells) in the presence of irradiated (30 Gy for 5 minutes) allogeneic PBMCs as feeder cells. Cells were cultured in Iscove modified Dulbecco medium (Gibco, ThermoFisher, UK) with 10% human serum (Sigma-Aldrich, USA) and 1% penicillin and streptomycin (Gibco, ThermoFisher, UK). Every 14 to 18 days, T cells were re-stimulated with fresh IL-2 and feeder cells.

### Lentiviral transduction of CTLs with wildtype RAB27A-GFP

Virus was produced after transfection of HEK293T cells with 6 μg pHR-SIN-RAB27A-GFP, 4 μg pMDG VSV-G (envelope plasmid) and 4 μg pCMV delta 8.9 (packaging plasmid) using TransIT transfection reagents (Mirrus). Virus containing supernatant was harvested after 48 hours and concentrated with LentiX concentration reagent (Clontech, Takara). CTLs were transduced with concentrated viral particles in the presence of 6 μg protamine sulfate (P4020-1G, MilliporeSigma).

### Western blot

Western blot was performed from CTLs as described previously (14). In brief: 2 × 10^7^ cells/ml were lysed in lysis buffer (50 mM Tris–HCl (pH7.4), 150 mM NaCl, 1% Triton X-100, 1% NP-40, 2 mM EDTA, 1x Halt™ protease inhibitors (ThermoFisher)) for 30 min on ice before cell debris was pelleted. Cell lysate was mixed with 4x NuPAGE™ LDS reducing sample buffer (Tris base (141 mM), Tris HCl (106 mM), LDS (2%), EDTA (0.51 mM), 50mM DTT) and heated at 95°C for 5 min prior to separation on a 4–12% NuPage™ Bis-Tris gel in MES running buffer (MES (50 mM), Tris base (50 mM), SDS (0.1%), EDTA (1 mM), pH 7.3 (all ThermoFisher)). To identify protein Plus Protein Kaleidoscope Prestained Protein Standards (Biorad) was used. Protein transfer onto nitrocellulose membranes (Trans-Blot^®^ Turbo™ Mini Nitrocellulose Transfer Packs (BioRad) was performed with a semi-dry Trans-Blot Turbo Transfer System (BioRad). Membranes were blocked in TBS, 5% non-fat dried milk and 0.05% Tween-20 (Sigma-Aldrich). Incubation of membrane with primary antibodies rabbit anti-RAB27A (Abcam) at 1:100 in blocking buffer at 4°C overnight or 1:5000 rabbit anti-actin (Sigma) for 1h at room temperature. Secondary antibody was 1:10,000 diluted goat anti-rabbit (H+L) HRP labelled (ThermoFisher). Membranes were developed using ECL Prime Western Blotting solution (Amersham) and imaged using a BioRad Chemidoc.

### Degranulation and cytotoxicity assay of CTL culture

CTLs were used at day10 of culture and were starved overnight without IL-2. CTLs were stimulated with plate-bound anti-CD3e (1μg/mL, UCHT1, BD Biosciences, UK) or with anti-CD3e (1μg/mL) coated P815 cells. Anti-CD107a (2μg/mL, Clone H4A3, eBioscience, UK) was added for the 3hrs stimulation time and cytotoxic T cells were stained with anti-CD8, Clone: MEM-31 (Abcam, UK) or 53-6.7 (BioLegend). Cells were analyzed with flow cytometry (Attune NxT, ThermoFisher, UK) and FlowJo software.

Cytotoxicity was measured as described before (36). In brief, cultured CTLs were stimulated with 1 μg/mL anti-CD3e antibody (clone UCHT1, BD Biosciences – Pharmingen) coated P815 cells, which express a NucLight Red dye. The used CTL-to-target ratio was 10:1. The killing was measured by the reduction of red fluorescence intensity, indicating target cell death. The assay was measured every 30 minutes for 15 hrs using the IncuCyte S3 live-cell analysis system (Essen Bioscience).

### Immunofluorescence microscopy

CTLs were washed and allowed to adhere for 12 minutes onto hydrophobic mutliwell slides (Hendles-Essex) in FCS-free IMDM. For conjugation assays, blue P815 target cells were coated with anti-CD3 (UCHT1), plated on multiwall slides in serum free media and CTLs were added for 10-15min. Cells were fixed with 4% paraformaldehyde (15710-S, Electron Microscopy Systems, USA) and incubated in quenching solution (50 mM ammonium chloride in 1X PBS) (15 min at RT) and rinsed twice in PBS. Permeabilization was done with 0.2% Saponin in PBS (5 min, RT) followed by washing and blocking for 30min in blocking buffer (1xPB, 1%BSA, 0.2% saponin). Cells were stained with anti-LAMP1 (H4A3, hybridoma supernatant), anti-RAB27A (abcam, UK) for 1 hour, followed by fluorophore-conjugated secondary antibodies (donkey anti-mouse 488 and goat anti-rabbit 647, Invitrogen, UK) (1 hour at RT) together with phalloidin 568 (Invitrogen, UK). Nuclei were stained for 5 minutes at RT with Hoechst (Thermo Fisher, UK), and samples were mounted in ProLong Diamond Antifade Reagent (Thermo Fisher, UK).

### Plasmid construction

The cDNA encoding mouse RAB27A(Asp7Tyr) was prepared by the standard molecular biology techniques using the following mutagenic oligonucleotide with a BamHI linker: 5’-CTAGGATCCATGTCGGATGGAGATTACTATTAC-3’. The RAB27A(Asp7Tyr) cDNA was subcloned into the pEF-FLAG tag expression vector (37) and pEGFP-C1 vector (Takara Bio Inc., Shiga Japan). The RAB27A(Asp7Tyr/Gln78Leu/Cys219Ala/Cys221Ala) cDNA was similarly prepared by PCR using pGBD-C1-Rab27A(Gln78Leu/Cys219Ala/Cys221Ala) (Gln78Leu: constitutively active mutation (38); as a template and subcloned into the pGBD-C1 vector (39). The mouse MLPH/SLAC2-A-SHD (Slp homology domain) (amino acids 1–153) and SLP2-A-SHD (amino acids 1–87) were subcloned into the pAct2 expression vector (Takara Bio Inc.). pEF-T7-MUNC13-4 and pEF-FLAG-RAB27A were prepared as described previously (37, 40).

### Melanosome distribution assays in melan-ash cells

Melan-ash cells, an immortal mouse melanocyte cell line derived from a RAB27A-deficient mouse (ashen mouse)(obtained from the Wellcome Trust Functional Genomics Cell Bank at St George’s, University of London), and were cultured as described previously (41). Transfection of pEGFP-C1 plasmids into melan-ash cells was achieved by using Lipofectamine^®^ 2000 (Thermo Fisher Scientific, Waltham, MA) according to the manufacturer’s instructions. Two days after transfection, the cells were fixed in 4% paraformaldehyde and examined for EGFP fluorescence with a confocal fluorescence microscope through an objective lens (×60 magnification, N.A. 1.40) (FluoView 1000-D, Evident/Olympus, Tokyo, Japan). Melanosome distribution assays were performed as described previously (42). The protein expression level of EGFP-tagged proteins in melan-ash cells was confirmed by immunoblotting with anti-GFP rabbit polyclonal antibody (MBL, Nagoya, Japan) and anti-β-actin-HRP mouse monoclonal antibody (Proteintech, Rosemont, IL).

### Binding assays

Co-immunoprecipitation assays in COS-7 cells and immunoblotting were performed as described previously (14). Anti-FLAG M2 magnetic beads (Sigma-Aldrich, St. Louis, MO), horseradish peroxidase (HRP)-conjugated anti-FLAG mouse monoclonal antibody (Sigma-Aldrich), and HRP-conjugated anti-T7 mouse monoclonal antibody (Merck Biosciences Novagen, Darmstadt, Germany) were obtained commercially. Yeast two-hybrid assays were also performed as described previously (38, 39). In brief, yeast cells expressing pGBD-C1-RAB27A (Gln78Leu/Cys219Ala/Cys221Ala) (WT or Asp7Tyr) plasmids and pAct2-MLPH/SLAC2-A-SHD or pACT2-SLP2-A-SHD plasmids were streaked on a growth medium (a synthetic complete medium lacking leucine and tryptophan: SC-LW) and on a selection medium (a synthetic complete medium lacking adenine, histidine, leucine, and tryptophan: SC-AHLW) and incubated two days and four days, respectively.

## Results

### Clinical susceptibility to severe EBV infections and lymphoma

**Figure 1:**
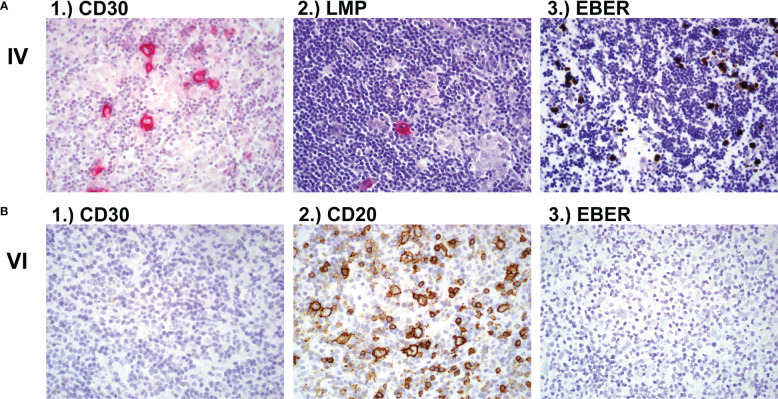
Lymph node biopsy **(A)** Lymph node biopsy of family member IV with classic Hodgkin lymphoma showing 1.) CD30 positive cells and 2.) LMP-EBV positive cells after hematoxylin and eosin (H&E) stain and 3.) EBER positive cells after *in-situ* hybridisation. **(B)** Lymph node histology of family member VI showing 1.) CD30 negative, 2.) CD20 positive and 3.) EBV negative NLPHL (nodular lymphocyte predominant Hodgkin lymphoma). LMP, latent membrane protein; EBV, Epstein-Barr Virus; EBER, Epstein-Barr virus (EBV)-encoded small RNAs.

The affected siblings of consanguineous parents presented with susceptibility to severe EBV infections and EBV-driven development of lymphoma (2/7 children). Patient IV was diagnosed with EBV-associated Hodgkin lymphoma with CD30+ cells (**Figure 1A**). At the time of diagnosis, EBV was detected by PCR in a bronchioalveolar lavage (16469 copies/μg) and blood (604 copies/μg). The patient received stem cell transplantation from his HLA-matched brother (VIII) after his fifth relapse of lymphoma. At the time of transplantation, genetic analysis of patient VIII had not been performed. The patient (IV) died of post-transplant lymphoproliferative EBV-driven disorder (PTLD) at 21 years of age. Patient VI developed CD20+ NLPHL (nodular lymphocyte predominant Hodgkin lymphoma) at 17 years of age, which in contrast to his brother’s lymphoma, was negative for CD30 and EBV antigens (**Figure 1B**). The patient is in remission after radiotherapy and surgical resection (R0). The youngest sister IX developed EBV-positive chronic-hyperplastic tonsillitis, which required a tonsillectomy due to sleep apnea. There was no sign of malignancy observed in the histological analysis. Her lymph nodes showed presence of abundant neutrophils. Normal T and B cell distribution was seen in the tonsils via flow cytometry. Brother V was EBV seropositive without any clinical manifestation. Patient VIII was PCR negative and serological investigation did not show a history of EBV infection until the age of 13.

### Metabolic manifestations

**Figure 2:**
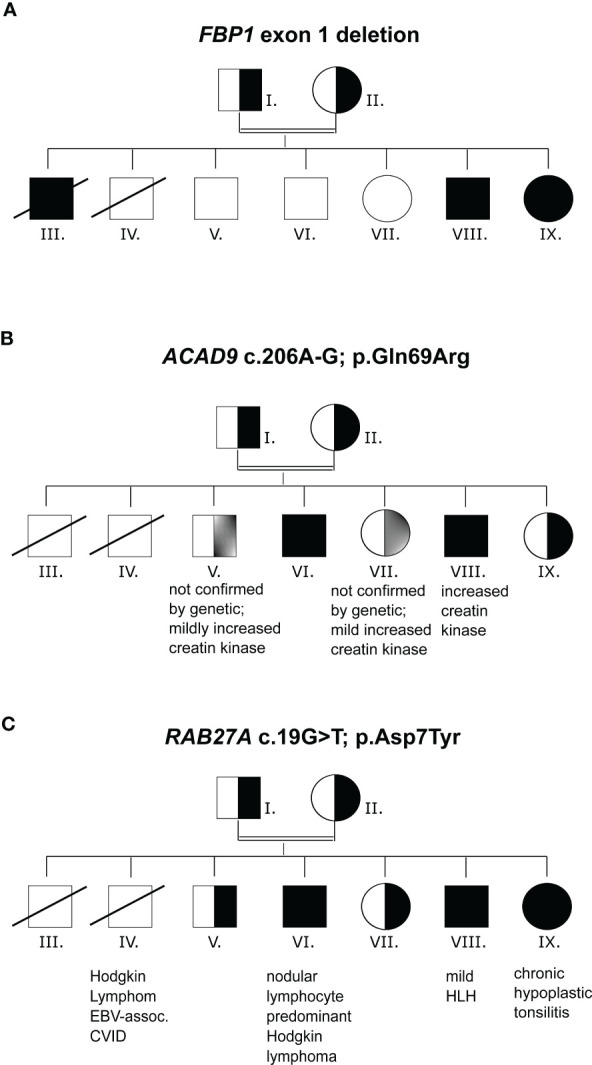
Mutation analysis **(A)** Family pedigree with *FBP1*, exon1 deletion inheritance. **(B)** Family pedigree with *ACAD9* c.206A-G inheritance. **(C)** Family pedigree with *RAB27A* c.19G>T inheritance. n.a., not analyzed.

Patient (VIII) showed a complex clinical phenotype including metabolic abnormalities with increased creatine kinase (827- 3942 U/L) and myopathy, abdominal pain and hypoglycemia. The patient had suffered thus far from five episodes of lactic acidosis (1.9-7.74mmol/L), abdominal pain, hypoglycemia and encephalopathy. At the age of 8 years, a muscle biopsy revealed a pathological increase of intracellular lipid droplets. The patient suffered from severe muscle pain after 2 hours of normal physical activity. Aspartate-aminotransferase (GOT) was always slightly increased, measuring 50-73 U/L. The forearm ischemia test was pathological (lactate increase from 1.9mmol/L to 4mmol/L). During catabolic episodes (e.g. infection-associated lung inflammation with respiratory insufficiency) at the ages of 9 and 13 years, the patient presented with abdominal pain, vomiting and loss of consciousness. Laboratory results showed hypoglycemia, increased lactate (7.74mmol/L) and increased pyruvate (0.22mmol/L). The episode at the age of 13 years led to hemophagocytic lymphohistiocytosis (HLH). A urine sample showed increased levels of lactate and pyruvate, as well as proline and alanine. Two genetic variants potentially associated with inborn errors of metabolism were detected: Fructose-1,6-biphosphatase (FBP1) deficiency was diagnosed based on a homozygous deletion of exon 1 of *FBP1* (*FBP1^m/m^*, NM_000507.4), which contains the translation start codon (**Figure 2A** and **Table 1**). Deletion of exon 1 and other FBP1 deletions have been described previously in cases of FBP1 deficiency (43). A diagnosis of ACAD9 deficiency was made based on the detection of a homozygous missense mutation in the acyl-CoA dehydrogenase gene *ACAD9* (NM_014049.5; c.206A>G; p.(Gln69Arg), *ACAD9^m/m^)* (**Figure 2B** and **Table 1**). This variant is exceedingly rare (allele frequency 0.000016 according to gnomAD) and was classified as deleterious or likely pathogenic by most prediction algorithms including SIFT, PolyPhen-2, REVEL and MetaLR. CADD scores categories this variant in the 0.5% most deleterious single nucleotide variants (score 23.4). Mutations in either of these genes can result in abdominal pain, hypoglycemia and lactic acidosis (44). The homozygous *FBP1* deletion was also found in sister IX, whose main clinical manifestations were episodes of hypoglycemia and increased lactate, ranging from 2.7-7.8 mmol/L. Additionally, abdominal pain was reported for brother III by family members, and we suspect that the same *FBP1* homozygous deletion or *ACAD9* may have been present. This patient died at the age of 1.5 years, in the context of complicated otitis media and hypoglycemia. No DNA was available for genetic confirmation. The homozygous *ACAD9* variant was also found in brother VI, who was reportedly suffering from catabolic episodes. A heterozygous *ACAD9* variant was assumed to underlie the mildly increased creatine kinase levels and myopathy of brothers V and VII, but no genetic analysis could be performed.

### Hypomorphic *RAB27A* missense mutation without albinism

**Figure 3:**
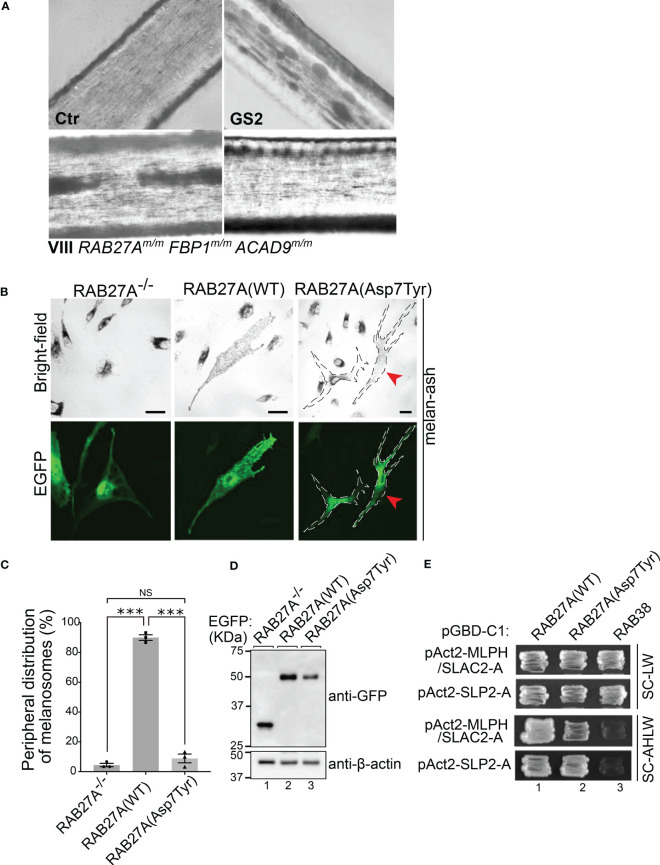
Analysis of RAB27A (Asp7Tyr) effects in hair and cultured melanocytes **(A)** Light microscopy of hair pigment distribution of a control (Ctr), patient with Griscelli syndrome Type 2 (GS2, *RAB27A*-deficiency) and patient VIII. **(B)** Typical images of RAB27A-deficient melan-ash cells transiently expressing EGFP alone, EGFP-RAB27A(WT), or EGFP-RAB27A(Asp7Tyr). EGFP-RAB27A (Asp7Tyr)-expressing cells in the right panels were outlined with dotted lines. Red arrowheads indicate rescued cells with normal peripheral melanosome distribution. Scale bars, 20μm. **(C)** Percentages of cells showing a peripheral melanosome distribution. Error bars = Mean ± S.E. of three independent experiments (n > 25 cells in each experiment). *** = p < 0.001; NS, not significant (one-way ANOVA and Tukey’s test). **(D)** Protein expression levels of EGFP, EGFP-RAB27A(WT) and EGFP-RAB27A(Asp7Tyr) in melan-ash cells by immunoblotting with antibodies indicated. **(E)** Interaction of RAB27A(WT) or RAB27A(Asp7Tyr) with MLPH/SLAC2-A-SHD or SLP2-A-SHD by yeast two-hybrid assays. RAB38, a melanosomal protein that does not bind to RAB27A effectors was used as a negative control (45).

The *FBP1* and *ACAD9* mutations alone did not explain the susceptibility to severe EBV infections. Whole exome sequencing revealed a previously unknown homozygous mutation in *RAB27A* (c.19G>T, p.(Asp7Tyr), *RAB27A^m/m^*) in patients VI, VIII and IX and heterozygosity in the remaining family members (**Figure 2C** and **Table 1**). The affected family members did not show any macroscopic signs of hair hypopigmentation or any characteristic pigment accumulations, which are usually seen in the hair shaft of GS2 patients (**Figure 3A**). As described above, GS2 patients often develop the life-threatening syndrome hemophagocytic lymphohistiocytosis (HLH) which is an indication for stem cell transplantation.

At 13 years of age, patient VIII suffered a flare of HLH, which could be controlled by treatment with corticosteroids, etoposide, and cyclosporine A. Six months later, he underwent stem cell transplantation from a matched (10/10) unrelated donor, after conditioning with treosulfan, fludarabin, thiotepa, and ATG. Post transplantation, the patient experienced acute GvHD IV° (including gut, liver, and skin) and chronic extensive GvHD of the skin, which was resolved by immunosuppressive treatment. Patient IX, who also harbours the RAB27A mutation and EBV infection, has not experienced any HLH symptoms up to the time of writing and the family has so far decided against stem cell transplantation.

### p.(Asp7Tyr) in *RAB27A* only partially mediates peripheral melanosome distribution in cultured melanocytes and showed reduced MLPH (melanophilin) binding activity

To further investigate whether the mutation p.(Asp7Tyr) in RAB27A affects melanosome distribution in cultured melanocytes, an enhanced green fluorescent protein (EGFP)-tagged Asp7Tyr mutant or wild-type (WT) RAB27A were transiently expressed in RAB27A deficient melan-ash cells. The melan-ash cells normally present with black mature melanosomes aggregated in the perinuclear region (**Figure 3B**) (40, 41). Re-expression of RAB27A (WT) in melan-ash cells completely restored peripheral melanosome distribution (**Figure 3B**). When RAB27A (Asp7Tyr) was expressed ~90% of the cells still exhibited a perinuclear aggregation phenotype (**Figure 3B**, right panels). Interestingly, ~10% of the RAB27A (Asp7Tyr)-expressing cells, presented with restored peripheral melanosome distribution (**Figure 3B**, red arrowheads in the right panels, and **Figure 3C**). This is in contrast to other RAB27A non-albinism causing mutations e.g., p.Lys22Arg, where a cytosolic localization of mutant proteins is observed (40). Under our experimental conditions, the attenuation of the perinuclear aggregation phenotype observed after transient expression of RAB27A(Asp7Tyr) was not statistically significant (**Figure 3C**). However, RAB27A(Asp7Tyr) protein expression was lower than that of RAB27A(WT), by at least 2-fold, which may mask the full effects of RAB27A(Asp7Tyr) re-expression (**Figure 3D**, lanes 2 and 3).

We performed yeast two hybrid assays in melanocytes, to investigate whether the Asp7Tyr mutation affects RAB27A’s ability to bind to melanophilin (MLPH, also known as SLAC2-A) and SLP2-A, both being known interaction partners of RAB27A (45–47) (**Figure 3E**, SC-AHLW panels). These assays demonstrated normal binding of RAB27A(Asp7Tyr) to SLP2-A but only a weak interaction with MLPH. Tyr-7 is a highly conserved residue in both invertebrate and vertebrate RAB27 (48), and previous structural analyses of RAB27 in a complex with its effectors indicated that Tyr-7 of RAB27 is located in one interphase of the RAB27–effector interaction sites (49, 50). Thus, the reduced MLPH binding activity of RAB27A(Asp7Tyr) could be caused by disruption of one of the RAB27–MLPH interaction sites. SLP2-A has been shown to bind to RAB27A with ten-fold higher affinity (KD = ~ 20 nM) than MLPH (KD = ~ 200 nM) (51), thus the Asp7Tyr mutation may be of little consequence on the RAB27A–SLP-2A interaction.

### RAB27A (Asp7Tyr) mutation leads to reduced lytic granule exocytosis and impaired CD8 T cell mediated killing *in vitro*, despite normal polarization at the immunological synapse

**Figure 4:**
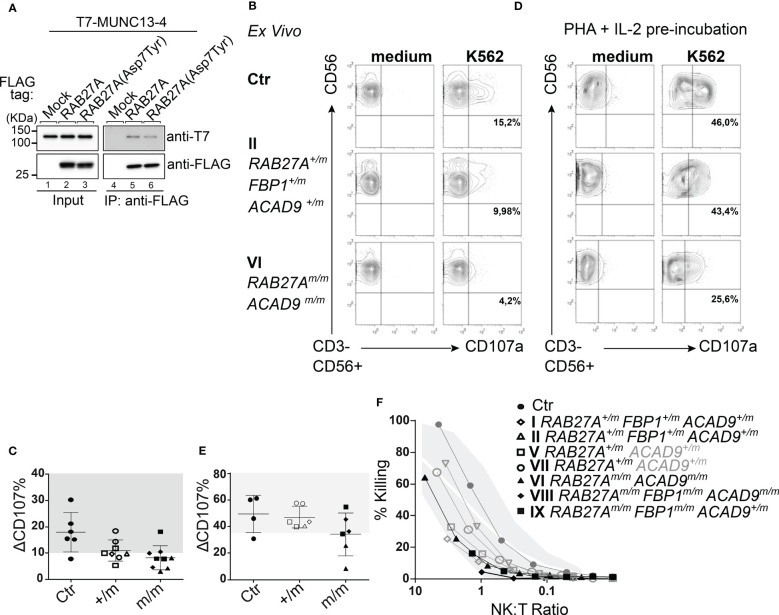
Effect of RAB27A (Asp7Tyr) mutation on NK cell degranulation **(A)** Co-immunoprecipitation of T7-MUNC13-4 and FLAG-RAB27A (WT or Asp7Tyr) in COS-7 cells, using anti-FLAG M2 magnetic beads. Co-immunoprecipitated T7-MUNC13-4 and immunoprecipitated FLAG-RAB27A were detected by immunoblotting with the antibodies indicated. **(B)** *Ex vivo* NK (CD3-CD56+) cells were either unstimulated in medium or stimulated with K562. Degranulation was measured by surface expression of CD107a. **(C)** Percent CD107a positive cells (normalized against medium only) relative to healthy controls (gray area). **(D)** NK cell degranulation of pre-activated NK cells (PHA (1μg/ml) and IL-2 (200U/ml) for 72 hrs), with either medium alone or K562 cells. **(E)** Percent CD107a positive pre-activated (as in D) NK cells (normalized against medium only) relative to healthy controls (gray area). **(F)** NK cell cytotoxicity measured by Cr^51^ release assay after 4hrs incubation. m, mutated; +, wildtype.

We investigated the interaction between RAB27A(Asp7Tyr) and MUNC13-4 - necessary for lytic granule priming and fusion - by co-immunoprecipitation assays in COS-7 cells. MUNC13-4 interaction with RAB27A(Asp7Tyr) was decreased by ~50% as compared to RAB27A(WT) (**Figure 4A**).

**Figure 5:**
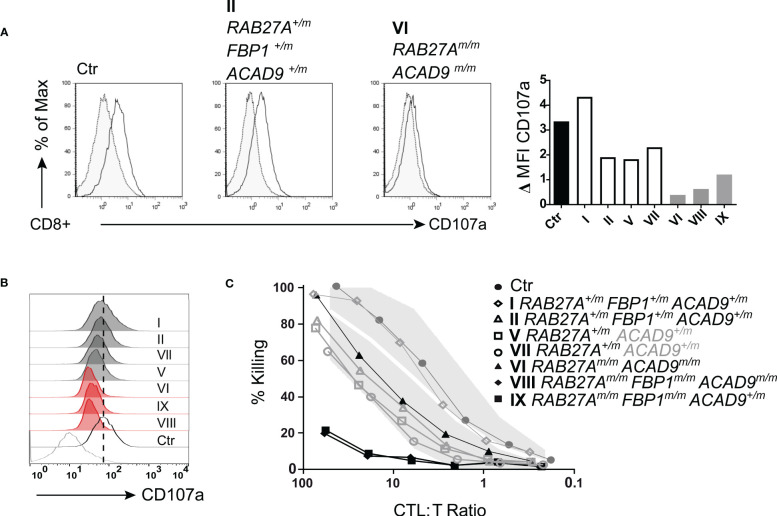
T cell degranulation and cytotoxicity **(A)** T cell degranulation analyzed following a 48 hr pre-stimulation with PHA (1μg/ml) and IL-2 (200U/ml). Cultured T cells were incubated in medium or stimulated with CD3/CD28 beads. CD107a expression upon stimulation. Gray area/dotted line = unstimulated, transparent area/solid line = stimulated. **(B)** Histogram summary of degranulation of homozygous RAB27A-deficient patients (red) and heterozygous RAB27A-carriers (grey) in comparison to a healthy control (black) and medium (bottom grey line). **(C)** Pre-activated (7 days with PHA (1μg/ml) and IL-2 (200U/ml)) T cell cytotoxicity measured by Cr^51^ release assay after 4hrs incubation with target cells. m, mutated; +, wildtype.

We then investigated the exocytosis of lytic granules and cytotoxic function of freshly isolated NK cells. Family members with homozygous RAB27A(Asp7Tyr) (VI, VIII, IX) showed reduced (<10%) lytic granule exocytosis, as measured by CD107a surface expression after stimulation with K562 target cells. NK cells from the RAB27A(Asp7Tyr) heterozygous siblings (V and VII who did not present with any clinical manifestations or genetic alterations pointing to FBP1-deficiency) and parents (I and II) showed normal to slightly reduced lytic granule exocytosis/degranulation (**Figures 4B, C**). After pre-activation of NK cell with PHA/IL-2, exocytosis of CD107a was restored to normal levels for affected siblings VIII and IX (**Figures 4D, E**), whereas the NK cells of VI still showed reduced degranulation (**Figures 4D, E**). NK cell killing capacity after a 4hr co-culture with K562 target cells was reduced for VIII (*RAB27A^m/m^, ACAD9^m/m^, FBP1^m/m^)*. NK cells from IX (*RAB27A^m/m^, FBP1^m/m^)* also showed reduced killing, whereas NK cell killing in VI (*RAB27A^m/m^, ACAD9^m/m^)* was normal. Family members heterozygous for RAB27A(Asp7Tyr) had normal NK cell killing (**Figure 4F**). Similar phenotypes were seen after pre-activation of CD8 T cells with PHA and IL-2: lack of exocytosis (as measured by CD107a surface expression) after stimulation in RAB27A homozygous and reduced exocytosis in heterozygous family members (**Figures 5A, B**). Longer culture under PHA/IL-2 conditions (7-9 days) showed that the RAB27A heterozygous CD8+ T cells from I, II, V and VII were able to kill target cells after 4hrs co-culture with target cells, as well as from patient VI with the homozygous *RAB27A* mutation. Remarkably, patient VIII and IX showed no CD8+ T cell cytotoxicity. Both patients were also homozygous for the *FBP1* mutation, which might influence CD8 T cell serial killing capacity in short-term cultured CD8 T cells re-stimulated with target cells (**Figure 5C**).

**Figure 6:**
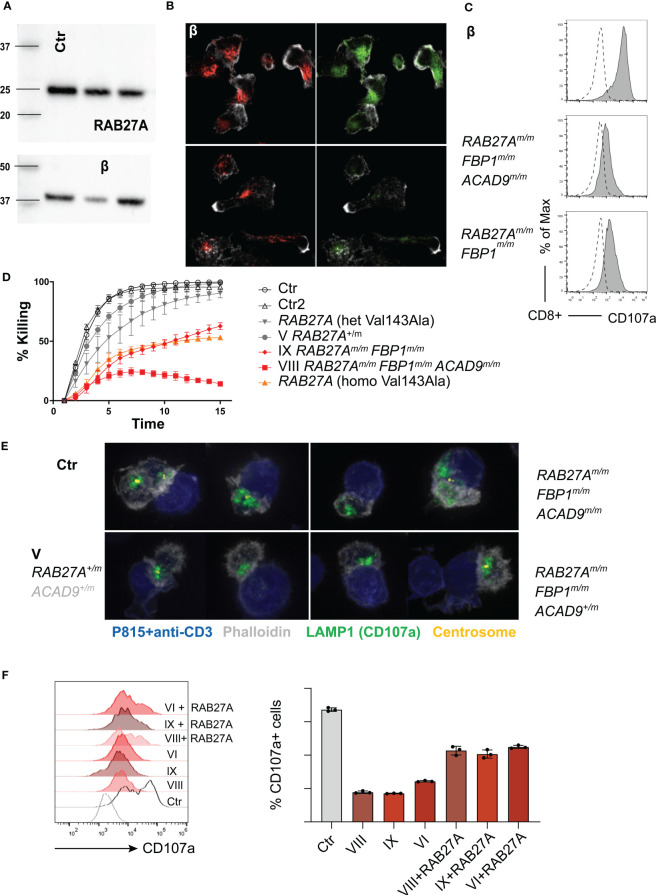
Degranulation, cytotoxicity and granule polarization of T cell lines **(A)** Representative western blot (n=3) of RAB27A protein expression levels in control or patient derived T cell lines 10 days after re-stimulation. **(B)** Representative immunofluorescence microscopy of T cell lines: RAB27A (green) LAMP1 (red), actin (phalloidin in grey) (n=2). **(C)** Degranulation in cytotoxic T cell lines post stimulation with medium (dotted line) or 1μg/ml plate bound anti-CD3 (grey, solid line) measured by CD107a staining in culture. **(D)** Cytotoxicity was analyzed by killing of red-fluorescent target cells, visualized using an Incucyte microscope Controls and RAB27A Val431Ala patients were described before (14). **(E)** Granule polarization by immunoflourescence staining. anti-CD3 coated target cells (blue), granules (anti-LAMP1/CD107a, green), actin (phalloidin, grey) and centrosome (yellow). **(F)** Representative granule exocytosis of cytotoxic T cells from patients VIII, VI (n=3) and IX (n=1). T cells were virally transduced with RAB27A-GFP or left untreated. CD107a is shown of CD8+RAB27A-GFP+ cells or CD8+ cells after stimulation with anti-CD3 coated P815 cells. m, mutated; +, wildtype.

To further evaluate RAB27A protein expression in CD8 T cells, we generated cytotoxic T cell (CTL) lines, by stimulation with allogeneic PBMCs, PHA and IL-2 every 3-4 weeks. To reduce the effects of IL-2 supplementation, which can increase granule exocytosis (35, 52), we starved the CTLs of IL-2 for 16 hours before performing the assays. Expression of mutant RAB27A, as measured by Western blot, was slightly reduced in the CTLs in comparison with the control sample (**Figure 6A**). Consistently, immunofluorescence staining demonstrated reduced levels of RAB27A (green), while distribution of lytic granules (LAMP1, red) was normal and co-localized with RAB27A (**Figure 6B**). Degranulation assays with cultured CTLs from homozygous *RAB27A*-variant family members, revealed impaired granule exocytosis as seen by presentation of CD107a/LAMP1 on the cell surface, after stimulation with plate-bound anti-CD3 (**Figure 6C**). Additionally, cytotoxicity in CTLs was measured by killing of red-fluorescently labelled target cells over a time period of 15 hours using an Incucyte microscope. We observed slower to normal killing by heterozygous (V) and impaired to absent killing by homozygous *RAB27A*-deficient patient-derived cells (IX and VIII respectively) (**Figure 6D**). Of note, patient VIII’s CTLs (*RAB27A^m/m^, ACAD9^m/m^, FBP1^m/m^)* presented with a total lack of killing activity. To understand if lytic granules are affected in their polarization, we stained conjugates of anti-CD3-labelled P815 target cells (blue) with CTLs using anti-LAMP1 (green) for lytic granules, phalloidin for actin (grey) and anti-pericentrin for centrosome (yellow). Successful formation of an immunological synapse is judged on the level of actin clearance and localization of the centrosome at the cell-to-cell contact site. Lytic granules polarized normally in all patients regardless of *RAB27A* mutations, with the notable exception of patient VIII, who is the only family member with compounding homozygous *FBP1* and *ACAD9*-deficiency (**Figure 6E**). Viral transduction and reconstitution of CTLs from patient VI, VIII an IX with wild type RAB27A-GFP only partially rescued the exocytosis defect of lytic granules (**Figure 6F**). In summary, the RAB27A(Asp7Tyr) variant seems to impair cytotoxic function of NK cells and T cells. Siblings VIII and IX showed no CD8+ T cell cytotoxicity. Both patients also had homozygous mutation in *FBP1*, which might influence CD8 T cell serial killing capacity in short-term cultured CD8 T cells. In long-term cultured cytotoxic T cell lines, the killing capacity of patient IX’s CTLs was reduced and comparable to CTLs from a previously described homozygous RAB27A (Val143Ala) variant (14). Highly impaired serial-killing and impaired lytic granule polarization was only observed in CTLs from patient VIII, homozygous for the RAB27A(Asp7Tyr) variant, FBP1-deletion and ACAD9 variant (**Tables 1**, **2**).

**Table 2:** Immune phenotype.

PAT	NK cell degran *ex vivo*	NK cell degran +PHA/IL-2	NK cell cytotox.	T cell degran +PHA/IL-2	T cell cytotox.	Mutation	Degran	Cytotox	Granule polarization
III	ND	ND	ND	ND	ND	ND	ND	ND	ND
IV	ND	ND	ND	ND	ND	ND	ND	ND	ND
V	9.0 [5.29-11.8]	41.64	normal	reduced	normal	*RAB27A* ^+/m^	normal	normal	yes
VI	5.1 [2.9-8,2]	17.04 [8.5-25.6]	normal	absent	normal	*RAB27A* ^m/m^ *ACAD9 ^m/m^*	absent-reduced	ND	ND
VII	13.7 [8.4-18.4]	57.3	normal	reduced	normal	*RAB27A* ^+/m^	ND	ND	ND
VIII	8.3 [4.6-10.4]	40.8	reduced	absent	absent	*RAB27A* ^m/m^ *FBP1* ^m/m^ *ACAD9 ^m/m^*	absent	absent	no
IX	11.2 [7.8-18.1]	44.6	reduced	absent	reduced	*RAB27A* ^m/m^ *FBP1* ^m/m^ *ACAD9 ^+/m^*	absent	reduced	yes

ND, not done.

## Discussion

Management of patients with multiple genetic defects and a complex family history can be highly challenging. Here we demonstrate overlapping clinical and functional phenotypes of mutations in three different coding genes, which did not only affect the function of the immune system, but also caused inborn errors of metabolism. We describe a novel hypomorphic RAB27A variant, with ~50% reduced MUNC13-4 binding, impaired NK and CD8 T cell killing and reduced MLPH binding.

Cytotoxic activity of immune cells is important for elimination of infected or malignant cells (53, 54). If the cytotoxic machinery is impaired, neoplastic cells cannot be effectively eliminated (55, 56). However, primary HLH usually occurs early in life, when the risk of cancer development is low (57, 58). Several reports of lymphoma in patients with hypomorphic cytotoxicity defects, e.g. in *PRF1* or *UNC13D*, imply a predisposition to lymphoma (16, 53, 59–63). In line with this observation, it has been described that *Prf1*-deficiency increases the frequency of lymphoma development in murine lymphoma models (60, 64, 65). Additionally, studies of heterozygous family members of FHL-patients, demonstrated higher incidence of lymphoma (66). A few cases with hypomorphic *RAB27A* mutations and lymphoma have already been described (15, 16). Here, we demonstrate impaired cytotoxic function in family members harbouring a hypomorphic c.19G>T variant in *RAB27A*, leading to reduced EBV control and predisposition to lymphoma. We further hypothesize that lymphoma susceptibility might be aggravated by the additional mutations in *FBP1* and *ACAD9*, affecting energy metabolism and – potentially – killing activity in NK cells and CTLs.

Numerous studies of cellular metabolic changes in immune cells demonstrated altered immune cell function and exhausted immune cell phenotypes (67–72). Cong et al (73) investigated the role of FBP1 in NK and CD8 T cell function and demonstrated that increased levels of FBP1 limit glycolysis, which is important for NK and CD8 T cell effector function (73). The family members with a homozygous *FBP1* variant presented with lactic acidosis and hypoglycemia, a sign of reduced *FBP1* function (29, 32). Since serial killing by NK and CD8 T cells in both patients with compound *RAB27A* variant and *FBP1-*deficiency was reduced, we cannot exclude an additive effect of FBP1-deficiency on NK and CD8 T cell function. Additionally, exocytosis of lytic granules could only be partially restored in patient IX with the homozygous FBP1 variant, suggesting a potential role for FBP1 in that pathway. It is also likely that reduced FBP1 and therefore increased glycolysis increases the risk of tumor progression in certain cancer types (74–76).

Acyl-CoA dehydrogenase 9 (ACAD9) is involved in fatty acid oxidation and is critical for functional mitochondrial complex I assembly. The complex I is important for NADH re-oxidation and the establishment of the proton gradient necessary for ATP synthesis, by pumping protons out of the mitochondrial matrix. Lisci et al. (77) examined CD8 T cells defective in USP30, which controls mitophagy. USP30 knockout in CTLs resulted in decreased oxidative phosphorylation, disruption of mitochondria after naïve T cell receptor activation and impaired serial killing. They demonstrated that *de novo* synthesis of cytolytic proteins depends on intact mitochondrial translation (77). We observed highly impaired killing capability and reduced polarization of lytic granules to the immunological synapse in patient VIII (*RAB27A^m/m^, ACAD9^m/m^, FBP1^m/m^)*. In a *RAB27A*-deficiency context, lytic granules are blocked at the immunological synapse, unable to fuse with the plasma membrane, similar to our observations in the CTLs of patient IX (*RAB27A^m/m^, FBP1^m/m^).* Therefore, we hypothesize that the additional homozygous *ACAD9* mutation in patient VIII may impair vesicle trafficking, since the LAMP1+ lytic granules were distributed in the cytoplasm and not localized at the immunological synapse with the centrosomes. However, reconstitution of RAB27A in CTLs of patient VI (*RAB27A^m/m^*, *ACAD9^m/m^*) only partially rescued the lytic granule exocytosis defect. Unfortunately, lytic granule distribution could not be analyzed in VI. In line, recent reports demonstrated that acyl-CoA produced by mitochondria and the flux from the cytosol to the endoplasmatic reticulum determines the quality of the secretory pathway (78).

In summary, we described a unique phenotype, resulting from variants in key genes regulating both energy metabolism and vesicle trafficking. The more severe the genotype (variants in both energy metabolism and vesicle trafficking), the more severe the observed immune and clinical phenotype. Even though no clear genotype-to-phenotype correlation could be established in this family, we found impaired CD8 T cell killing, resulting in high vulnerability to viral infections and malignancies. We believe that with growing numbers of whole exome and whole genome analyses performed on patient samples, we will likely discover more patients with multiple variants, making the dissection of phenotypes more complex. Functional analysis of individual pathogenic or likely pathogenic variants detected by high-throughput sequencing is very time consuming. This family impressively showed that genetic analyses of variants - especially with combined effects leading to a complex genotype – can be supplemented by broader functional analyses, that - relatively quickly - assay systems and immune processes (e.g. NK and CTL granule exocytosis), as clinicians are forced to make fast treatment decisions, including indication for stem cell transplantation. We firmly believe that such methods can be invaluable in offering patients the most appropriate treatment and assist in deciding for or against stem cell transplantation.

## Data availability statement

The patient-derived genomic datasets presented in this article are not readily available for legal reasons; individual genomic DNA data cannot be made available.

## Ethics statement

The studies involving human participants were reviewed and approved by University of Freiburg ethics committee’s protocol numbers 143/12 and 40/08. Written informed consent to participate in this study was provided by the participants’ legal guardian/next of kin.

## Author contributions

Patient care and clinical workup: NB, SB, KL, TN. Resources (reagents and patient material): NP, WK, SE, GG, TN. Investigation and methodology: YM, KS, IO, HN, ML, LB, HH, SA. Writing original draft: SA. Writing – review & editing: TN, SE, HH, MF, SA. All authors contributed to the article and approved the submitted version.

## References

[1] Tangye SG Al-Herz W Bousfiha A Cunningham-Rundles C Franco JL Holland SM. Human Inborn Errors of Immunity: 2022 Update on the Classification from the International Union of Immunological Societies Expert Committee. J Clin Immunol (2020) 42:1473–507. doi: 10.1007/s10875-022-01289-3 PMC9244088 35748970

[2] Janka GE Lehmberg K. Hemophagocytic syndromes–an update. Blood Rev (2014) 28:135–42. doi: 10.1016/j.blre.2014.03.002 24792320

[3] Ammann S Lehmberg K Zur Stadt U Klemann C Bode S. Effective immunological guidance of genetic analyses including exome sequencing in patients evaluated for hemophagocytic lymphohistiocytosis. J Clin Immunol (2017) 37:770–80. doi: 10.1007/s10875-017-0443-1 28936583

[4] Heeg M Ammann S Klemann C Panning M Falcone V Hengel H. Is an infectious trigger always required for primary hemophagocytic lymphohistiocytosis? lessons from *in utero* and neonatal disease. Pediatr Blood Cancer (2018) 65:e27344. doi: 10.1002/pbc.27344 30070073 PMC7168068

[5] Marsh RA. Epstein-Barr Virus and hemophagocytic lymphohistiocytosis. Front Immunol (2018) 8:1–9. doi: 10.3389/fimmu.2017.01902 PMC5766650 29358936

[6] Xu X Wang H Ju X Xiao P Xiao Y Xue H. Clinical presentation and outcome of pediatric patients with hemophagocytic lymphohistiocytosis in China: a retrospective multicenter study. Pediatr Blood Cancer (2017) 64:e26264. doi: 10.1002/pbc.26264 27781387

[7] Fukuda M. Versatile role of Rab27 in membrane trafficking: focus on the Rab27 effector families. J Biochem (2005) 137:9–16. doi: 10.1093/jb/mvi002 15713878

[8] Menasche G Pastural E Feldmann J Certain S Ersoy F Dupuis S. Mutations in RAB27A cause griscelli syndrome associated with haemophagocytic syndrome. Nat Genet (2000) 25:173–6. doi: 10.1038/76024 10835631

[9] Krzewski K Cullinane AR. Evidence for defective rab GTPase-dependent cargo traffic in immune disorders. Exp Cell Res (2013) 319:2360–7. doi: 10.1016/j.yexcr.2013.06.012 PMC3759575 23810987

[10] Stinchcombe J Barral D Mules E Booth S Hume A Machesky L. Rab27a is required for regulated secretion in cytotoxic T lymphocytes. J Cell Biol (2001) 152:825–33. doi: 10.1083/jcb.152.4.825 PMC2195783 11266472

[11] Elstak ED Neeft M Nehme NT Voortman J Cheung M Goodarzifard M. The munc13-4–rab27 complex is specifically required for tethering secretory lysosomes at the plasma membrane. Blood (2011) 118(6):1570–8. doi: 10.1182/blood-2011-02-339523 21693760

[12] Fukuda M Kuroda TS Mikoshiba K. Slac2-a/melanophilin, the missing link between Rab27 and myosin va: implications of a tripartite protein complex for melanosome transport. J Biol Chem (2002) 277:12432–6. doi: 10.1074/jbc.C200005200 11856727

[13] Cetica V Hackmann Y Grieve S Sieni E Ciambotti B Coniglio M. Patients with griscelli syndrome and normal pigmentation identify RAB27A mutations that selectively disrupt MUNC13-4 binding. J Allergy Clin Immunol (2015) 135:1310–8.e1. doi: 10.1016/j.jaci.2014.08.039 25312756 PMC4418747

[14] Ohishi Y Ammann S Ziaee V Strege K Groß M Amos CV. Griscelli syndrome type 2 sine albinism: unraveling differential RAB27A effector engagement. Front Immunol (2020) 11. doi: 10.3389/fimmu.2020.612977 PMC7758216 33362801

[15] Woodward K Shah R Benseler S Wei X Ng D Grossman J. Considering immunologic and genetic evaluation for HLH in neuroinflammation: a case of griscelli syndrome type 2 with neurological symptoms and a lack of albinism. Pediatr Blood Cancer (2020) 67:3–5. doi: 10.1002/pbc.28312 32459386

[16] Tesi B Rascon J Chiang S Burnyte B Löfstedt A Fasth A. A RAB27A 5′ untranslated region structural variant associated with late-onset hemophagocytic lymphohistiocytosis and normal pigmentation. J Allergy Clin Immunol (2018) 142:317–321.e8. doi: 10.1016/j.jaci.2018.02.031 29522846 PMC6034010

[17] Froesch ER. Disorders of fructose metabolism. Clin Endocrinol Metab (1976) 5:599–611. doi: 10.1016/S0300-595X(76)80042-4 189957

[18] Morris AAM Spiekerkoetter U. Disorders of Mitochondrial Fatty Acid Oxidation & Riboflavin Metabolism. In: Saudubray JM Baumgartner M Walter J (eds) Inborn Metabolic Diseases. Springer, Berlin, Heidelberg. (2016). doi: 10.1007/978-3-662-49771-5_12

[19] Schiff M Haberberger B Xia C Mohsen A Goetzman E Wang Y. Complex I assembly function and fatty acid oxidation enzyme activity of ACAD9 both contribute to disease severity in ACAD9 deficiency. Hum Mol Genet (2015) 24:3238. doi: 10.1093/hmg/ddv074 25721401 PMC4424958

[20] He M Rutledge S Kelly D Palmer C Murdoch G Majumder N. A new genetic disorder in mitochondrial fatty acid β-oxidation: ACAD9 deficiency. Am J Hum Genet (2007) 81:87–103. doi: 10.1086/519219 17564966 PMC1950923

[21] Repp BM Mastantuono E Alston C Schiff M Haack T Rötig A. Clinical, biochemical and genetic spectrum of 70 patients with ACAD9 deficiency: is riboflavin supplementation effective? Orphanet J Rare Dis (2018) 13:120. doi: 10.1186/s13023-018-0784-8 30025539 PMC6053715

[22] Scheffler IE. Assembling complex i with ACAD9. Cell Metab (2010) 12:211–2. doi: 10.1016/j.cmet.2010.08.008 20816087

[23] Leslie N. Neonatal multiorgan failure due to ACAD9 mutation and complex i deficiency with mitochondrial hyperplasia in liver, cardiac myocytes, skeletal muscle, and renal tubules. Hum Pathol (2016) 49:27–32. doi: 10.1016/j.humpath.2015.09.039 26826406

[24] Aintablian HK Narayanan V Belnap N Ramsey K Grebe TA. An atypical presentation of ACAD9 deficiency: diagnosis by whole exome sequencing broadens the phenotypic spectrum and alters treatment approach. Mol Genet Metab Rep (2016) 10:38–44. doi: 10.1016/j.ymgmr.2016.12.005 28070495 PMC5219625

[25] Sinsheimer A Mohsen A Bloom K Karunanidhi A Bharathi S Wu Y. Development and characterization of a mouse model for Acad9 deficiency. Mol Genet Metab (2021) 134:156–63. doi: 10.1016/j.ymgme.2021.09.002 PMC8588265 34556413

[26] Haack T Danhauser K Haberberger B Hoser J Strecker V Boehm D. Exome sequencing identifies ACAD9 mutations as a cause of complex I deficiency. Nat Genet (2010) 42:1131–4. doi: 10.1038/ng.706 21057504

[27] Steinmann B Santer R. Disorders of Fructose Metabolism. In: Saudubray JM Baumgartner M Walter J. (eds) Inborn Metabolic Diseases. Springer, Berlin, Heidelberg. (2016). doi: 10.1007/978-3-662-49771-5_8

[28] Pinheiro FC Ligabue-Braun R de Siqueira A Matuella C de Souza C Monteiro FP. The fructose-1,6-bisphosphatase deficiency and the p.(Lys204ArgfsTer72) variant. Genet Mol Biol (2021) 44. 10.1590/1678-4685-GMB-2020-0281 PMC8127874 33999094

[29] Pagliara AS Karl IE Keating JP Brown BI Kipnis DM. Hepatic fructose-1,6-diphosphatase deficiency. a cause of lactic acidosis and hypoglycemia in infancy. J Clin Invest (1972) 51:2115–23. doi: 10.1172/JCI107018 PMC292368 4341015

[30] Afroze B Yunus Z Steinmann B Santer R. Transient pseudo-hypertriglyceridemia: a useful biochemical marker of fructose-1,6-bisphosphatase deficiency. Eur J Pediatr (2013) 172:1249–53. doi: 10.1007/s00431-013-2084-6 23881342

[31] Kılıç M Kasapkara Ç.S Yılmaz DY Özgül RK. Exon 2 deletion represents a common mutation in Turkish patients with fructose-1,6-bisphosphatase deficiency. Metab Brain Dis (2019) 34:1487–91. doi: 10.1007/s11011-019-00455-8 31278438

[32] Baker L Winegrad AI. Fasting hypoglycaemia and metabolic acidosis associated with deficiency of hepatic fructose-1,6-diphosphatase activity. Lancet (1970) 2:13–6. doi: 10.1016/S0140-6736(70)92474-8 4193749

[33] Moey LH Abdul Azize N Yakob Y Leong H Keng W Chen B. Fructose-1,6-bisphosphatase deficiency as a cause of recurrent hypoglycemia and metabolic acidosis: clinical and molecular findings in Malaysian patients. Pediatr Neonatol (2018) 59:397–403. doi: 10.1016/j.pedneo.2017.11.006 29203193

[34] Salih RM Mohammed EA Alhashem AM Mohamed S Al-Aqeel AI. Fructose-1,6-bisphosphatase deficiency with confirmed molecular diagnosis. an important cause of hypoglycemia in children. Saudi Med J (2020) 41:199–202. doi: 10.15537/smj.2020.2.24885 32020156 PMC7841638

[35] Bryceson YT Pende D Maul-Pavicic A Gilmour K Ufheil H Vraetz T. A prospective evaluation of degranulation assays in the rapid diagnosis of familial hemophagocytic syndromes. Blood (2012) 119:2754–63. doi: 10.1182/blood-2011-08-374199 22294731

[36] Randzavola LO Strege K Juzans M Asano Y Stinchcombe J Gawden-Bone C. Loss of ARPC1B impairs cytotoxic T lymphocyte maintenance and cytolytic activity. J Clin Invest (2019) 129:5600–14. doi: 10.1172/JCI129388 PMC6877333 31710310

[37] Kuroda TS Fukuda M Ariga H Mikoshiba K. The slp homology domain of synaptotagmin-like proteins 1-4 and Slac2 functions as a novel Rab27A binding domain. J Biol Chem (2002) 277:9212–8. doi: 10.1074/jbc.M112414200 11773082

[38] Fukuda M Kanno E Ishibashi K Itoh T. Large Scale screening for novel rab effectors reveals unexpected broad rab binding specificity. Mol Cell Proteomics (2008) 7:1031–42. doi: 10.1074/mcp.M700569-MCP200 18256213

[39] James P Halladay J Craig EA. Genomic libraries and a host strain designed for highly efficient two-hybrid selection in yeast. Genetics (1996) 144:1425–36. doi: 10.1093/genetics/144.4.1425 PMC1207695 8978031

[40] Ohbayashi N Mamishi S Ishibashi K Maruta Y Pourakbari B Tamizifar B. Functional characterization of two RAB27A missense mutations found in griscelli syndrome type 2. Pigment Cell Melanoma Res (2010) 23:365–74. doi: 10.1111/j.1755-148X.2010.00705.x 20370853

[41] Ali BR Wasmeier C Lamoreux L Strom M Seabra MC. Multiple regions contribute to membrane targeting of rab GTPases. J Cell Sci (2004) 117:6401–12. doi: 10.1242/jcs.01542 15561774

[42] Kuroda TS Ariga H Fukuda M. The actin-binding domain of Slac2-a/Melanophilin is required for melanosome distribution in melanocytes. Mol Cell Biol (2003) 23:5245–55. doi: 10.1128/MCB.23.15.5245-5255.2003 PMC165717 12861011

[43] Santer R Du Moulin M Shahinyan T Vater I Maier E Muntau A. A summary of molecular genetic findings in fructose-1,6-bisphosphatase deficiency with a focus on a common long-range deletion and the role of MLPA analysis. Orphanet J Rare Dis (2016) 11:1–7. doi: 10.1186/s13023-016-0415-1 27101822 PMC4839065

[44] Tran C. Inborn errors of fructose metabolism. what can we learn from them? Nutrients (2017) 9:356. doi: 10.3390/nu9040356 28368361 PMC5409695

[45] Strom M Hume AN Tarafder AK Barkagianni E Seabra MC. A family of Rab27-binding proteins: melanophilin links Rab27a and myosin va function in melanosome transport. J Biol Chem (2002) 277:25423–30. doi: 10.1074/jbc.M202574200 11980908

[46] Wu XS Rao K Zhang H Wang F Sellers J Matesic L. Identification of an organelle receptor for myosin-va. Nat Cell Biol (2002) 4:271–8. doi: 10.1038/ncb760 11887186

[47] Kuroda TS Fukuda M. Rab27A-binding protein Slp2-a is required for peripheral melanosome distribution and elongated cell shape in melanocytes. Nat Cell Biol (2004) 6:1195–203. doi: 10.1038/ncb1197 15543135

[48] Yu E Kanno E Choi S Sugimori M Moreira J Llinás R. Role of Rab27 in synaptic transmission at the squid giant synapse. Proc Natl Acad Sci U.S.A. (2008) 105:16003–8. doi: 10.1073/pnas.0804825105 PMC2562534 18840683

[49] Chavas LMG Ihara K Kawasaki M Torii S Uejima T Kato R. Elucidation of Rab27 recruitment by its effectors: structure of Rab27a bound to Exophilin4/Slp2-a. Structure (2008) 16:1468–77. doi: 10.1016/j.str.2008.07.015 18940603

[50] Kukimoto-Niino M Sakamoto A Kanno E Hanawa-Suetsugu K Terada T Shirouzu M. Structural basis for the exclusive specificity of Slac2-a/Melanophilin for the Rab27 GTPases. Structure (2008) 16:1478–90. doi: 10.1016/j.str.2008.07.014 18940604

[51] Fukuda M. Distinct Rab27A binding affinities of Slp2-a and Slac2-a/melanophilin: hierarchy of Rab27A effectors. Biochem Biophys Res Commun (2006) 343:666–74. doi: 10.1016/j.bbrc.2006.03.001 16554019

[52] Bryceson YT Rudd E Zheng C Edner J Ma D Wood S. Defective cytotoxic lymphocyte degranulation in syntaxin-11–deficient familial hemophagocytic lymphohistiocytosis 4 (FHL4) patients. Blood (2007) 110:1906–15. doi: 10.1182/blood-2007-02-074468 PMC1976360 17525286

[53] Cetica V Sieni E Pende D Danesino C De Fusco C Locatelli F. Genetic predisposition to hemophagocytic lymphohistiocytosis: report on 500 patients from the Italian registry. J Allergy Clin Immunol (2016) 137(1):188–96.e4. doi: 10.1016/j.jaci.2015.06.048 PMC4699615 26342526

[54] Smyth M Thia K Street S MacGregor D Godfrey D Trapani J. Perforin-mediated cytotoxicity is critical for surveillance of spontaneous lymphoma. J Exp Med (2000) 192:755–60. doi: 10.1084/jem.192.5.755 PMC2193269 10974040

[55] Hanahan D Weinberg RA. Hallmarks of cancer: the next generation. Cell (2011) 144:646–74. doi: 10.1016/j.cell.2011.02.013 21376230

[56] Dunn GP Bruce AT Ikeda H Old LJ Schreiber RD. Cancer immunoediting: from immunosurveillance to tumor escape. Nat Immunol (2002) 3:991–8. https://doi.org/doi: 10.1038/ni1102-991. 12407406

[57] Allen M De Fusco C Legrand F Clementi R Conter V Danesino C. Familial hemophagocytic lymphohistiocytosis: how late can the onset be? Haematologica (2001) 86:499–503. 11410413

[58] Janka GE Lehmberg K. Hemophagocytic lymphohistiocytosis: pathogenesis and treatment. Hematol Am Soc Hematol Educ Program (2013) 2013:605–11. doi: 10.1182/asheducation-2013.1.605 24319239

[59] Löfstedt A Ahlm C Tesi B Bergdahl I Nordenskjöld M Bryceson YT. Haploinsufficiency of UNC13D increases the risk of lymphoma. Cancer (2019) 125:1848–54. doi: 10.1002/cncr.32011 PMC6593970 30758854

[60] Chia J Kim P Whisstock J Dunstone M Trapani J Voskoboinik I. Temperature sensitivity of human perforin mutants unmasks subtotal loss of cytotoxicity, delayed FHL, and a predisposition to cancer. Proc Natl Acad Sci U.S.A. (2009) 106:9809–14. doi: 10.1073/pnas.0903815106 PMC2701033 19487666

[61] Pagel J Beutel K Lehmberg K Koch F Maul-Pavicic A Rohlfs A. Distinct mutations in STXBP2 are associated with variable clinical presentations in patients with familial hemophagocytic lymphohistiocytosis type 5 (FHL5). Blood (2012) 119:6016–24. doi: 10.1182/blood-2011-12-398958 22451424

[62] Machaczka M Klimkowska M Chiang S Meeths M Müller M Gustafsson B. Development of classical hodgkin’s lymphoma in an adult with biallelic STXBP2 mutations. Haematologica (2013) 98:760–4. doi: 10.3324/haematol.2012.073098 PMC3640121 23100279

[63] Tesi B Chiang S El-Ghoneimy D Hussein A Langenskiöld C Wali R. Spectrum of atypical clinical presentations in patients with biallelic PRF1 missense mutations. Pediatr Blood Cancer (2015) 62:2094–100. doi: 10.1002/pbc.25646 26184781

[64] Bolitho P Street S Westwood J Edelmann W MacGregor D Waring P. Perforin-mediated suppression of b-cell lymphoma. Proc Natl Acad Sci USA (2009) 106:2723–8. doi: 10.1073/pnas.0809008106 PMC2650333 19196996

[65] Street S Hayakawa Y Zhan Y Lew A MacGregor D Jamieson A. Innate immune surveillance of spontaneous b cell lymphomas by natural killer cells and γδ T cells. J Exp Med (2004) 199:879–84. doi: 10.1084/jem.20031981 PMC2212720 15007091

[66] Löfstedt A Chiang S Onelöv E Bryceson YT Meeths M Henter J. Cancer risk in relatives of patients with a primary disorder of lymphocyte cytotoxicity: a retrospective cohort study. Lancet Haematol (2015) 2:e536–42. doi: 10.1016/S2352-3026(15)00223-9 26686408

[67] Biswas SK. Metabolic reprogramming of immune cells in cancer progression. Immunity (2015) 43:435–49. doi: 10.1016/j.immuni.2015.09.001 26377897

[68] Gardiner CM Finlay DK. What fuels natural killers? metabolism and NK cell responses. Front Immunol (2017) 8:2017.00367. doi: 10.3389/fimmu.2017.00367 28421073 PMC5376555

[69] O’Neill LAJ Kishton RJ Rathmell J. A guide to immunometabolism for immunologists. Nat Rev Immunol (2016) 16:553–65. doi: 10.1038/nri.2016.70 PMC5001910 27396447

[70] Donnelly RP Loftus R Keating S Liou K Biron C Gardiner C. mTORC1-dependent metabolic reprogramming is a prerequisite for NK cell effector function. J Immunol (2014) 193:4477–84. doi: 10.4049/jimmunol.1401558 PMC4201970 25261477

[71] Caro-Maldonado A Wang R Nichols A Kuraoka M Milasta S Sun L. Metabolic reprogramming is required for antibody production that is suppressed in anergic but exaggerated in chronically BAFF-exposed b cells. J Immunol (2014) 192:3626–36. doi: 10.4049/jimmunol.1302062 PMC3984038 24616478

[72] Dietl K Renner K Dettmer K Timischl B Eberhart K Dorn C. Lactic acid and acidification inhibit TNF secretion and glycolysis of human monocytes. J Immunol (2010) 184:1200–9. doi: 10.4049/jimmunol.0902584 20026743

[73] Cong J Wang X Zheng X Wang D Fu B Sun R. Dysfunction of natural killer cells by FBP1-induced inhibition of glycolysis during lung cancer progression. Cell Metab (2018) 28:243–255.e5. doi: 10.1016/j.cmet.2018.06.021 30033198

[74] Hirata H Sugimachi K Komatsu H Ueda M Masuda T Uchi R. Decreased expression of fructose-1,6-bisphosphatase associates with glucose metabolism and tumor progression in hepatocellular carcinoma. Cancer Res (2016) 76:3265–76. doi: 10.1158/0008-5472.CAN-15-2601 27197151

[75] Dong C Yuan T Wu Y Wang Y Fan T Miriyala S. Loss of FBP1 by snail-mediated repression provides metabolic advantages in basal-like breast cancer. Cancer Cell (2013) 23:316–31. doi: 10.1016/j.ccr.2013.01.022 PMC3703516 23453623

[76] Li B Qiu B Lee D Walton Z Ochocki J Mathew L. Fructose-1,6-bisphosphatase opposes renal carcinoma progression. Nature (2014) 513:251–5. doi: 10.1038/nature13557 PMC4162811 25043030

[77] Lisci M Barton P Randzavola L Ma C Marchingo J Cantrell D. Mitochondrial translation is required for sustained killing by cytotoxic T cells. Sci (1979) (2021) 374. doi: 10.1126/science.abe9977 34648346

[78] Dieterich IA Cui Y Braun M Lawton A Robinson N Peotter J. Acetyl-CoA flux from the cytosol to the ER regulates engagement and quality of the secretory pathway. Sci Rep (2021) 11:1–17. doi: 10.1038/s41598-021-81447-6 33479349 PMC7820588

